# Increased Proinflammatory Cytokine Production and Decreased Cholesterol Efflux Due to Downregulation of ABCG1 in Macrophages Exposed to Indoxyl Sulfate

**DOI:** 10.3390/toxins7083155

**Published:** 2015-08-14

**Authors:** Koji Matsuo, Suguru Yamamoto, Takuya Wakamatsu, Yoshimitsu Takahashi, Kazuko Kawamura, Yoshikatsu Kaneko, Shin Goto, Junichiro J. Kazama, Ichiei Narita

**Affiliations:** 1 Division of Clinical Nephrology and Rheumatology, Niigata University Graduate School of Medical and Dental Sciences, Niigata 951-8510, Japan; E-Mails: ko.matsu.notre@gmail.com (K.M.); tkywakamatsu@yahoo.co.jp (T.W.); yoshimitsu2003@gmail.com (Y.T.); kazukawamura@hotmail.com (K.K.); kanekoy@med.niigata-u.ac.jp (Y.K.); gotos@med.niigata-u.ac.jp (S.G.); jjkaz@med.niigata-u.ac.jp (J.J.K.); naritai@med.niigta-u.ac.jp (I.N.); 2 Division of Blood Purification Therapy, Niigata University Medical and Dental Hospital, Niigata 951-8520, Japan

**Keywords:** indoxyl sulfate, macrophage, chronic kidney disease, atherosclerosis

## Abstract

One of the possible causes of enhanced atherosclerosis in patients with chronic kidney disease (CKD) is the accumulation of uremic toxins. Since macrophage foam cell formation is a hallmark of atherosclerosis, we examined the direct effect of indoxyl sulfate (IS), a representative uremic toxin, on macrophage function. Macrophages differentiated from THP-1 cells were exposed to IS *in vitro*. IS decreased the cell viability of THP-1 derived macrophages but promoted the production of inflammatory cytokines (IL-1β, IS 1.0 mM: 101.8 ± 21.8 pg/mL *vs.* 0 mM: 7.0 ± 0.3 pg/mL, TNF-α, IS 1.0 mM: 96.6 ± 11.0 pg/mL *vs.* 0 mM: 15.1 ± 3.1 pg/mL) and reactive oxygen species. IS reduced macrophage cholesterol efflux (IS 0.5 mM: 30.3% ± 7.3% *vs.* 0 mM: 43.5% ± 1.6%) and decreased ATP-binding cassette transporter G1 expression. However, lipid uptake into cells was not enhanced. A liver X receptor (LXR) agonist, T0901317, improved IS-induced production of inflammatory cytokines as well as reduced cholesterol efflux. In conclusion, IS induced inflammatory reactions and reduced cholesterol efflux in macrophages. Both effects of IS were improved with activation of LXR. Direct interactions of uremic toxins with macrophages may be a major cause of atherosclerosis acceleration in patients with CKD.

## 1. Introduction

Cardiovascular disease (CVD) is a major cause of death in patients with chronic kidney disease (CKD). Cardiovascular (CV) events and mortality are known to increase with decreased glomerular filtration rate [1,2], and CV mortality is more than 5 times higher in patients undergoing dialysis than in the general population [3]. Although there are several traditional risk factors for CVD in the general population, only a few partially explain the remarkably high incidence of CVD in patients with CKD [4]. Lipid-lowering treatments with statins and HMG-CoA reductase inhibitors reduce CV events and mortality in the general population [5,6,7]. However, this effect is lower in patients with CKD, especially in those undergoing dialysis treatments [8,9,10]. Accordingly, some CKD-specific risk factors are related to the development of CVD in patients with CKD [11]. One possible factor is the accumulation of protein-bound uremic toxins, secondary to their limited removal with conventional dialysis treatment [12]. For example, high serum levels of indoxyl sulfate (IS), a representative protein-bound uremic toxin, are associated with higher CV mortality in patients with CKD [13]. In a mouse model, kidney damage accelerated atherosclerosis characterized by the deposition of IS in the lesion. However, absorbent oral charcoal ameliorated the accumulation of IS in the lesion and simultaneously reduced atherosclerosis [14]. This indicates that IS reacts directly with cells within atherosclerotic lesions; however, the underlying mechanisms are poorly understood. Inflammatory reactions and macrophage foam cell formation are well-known hallmarks of atherosclerosis aggravation. Therefore, in the present study, we examined the direct interaction of IS with macrophages to understand the mechanisms of CKD-induced acceleration of atherosclerosis.

## 2. Results

### 2.1. Cell Viability, Inflammation and Reactive Oxygen Species Production in Macrophages Exposed to IS

**Figure 1:**
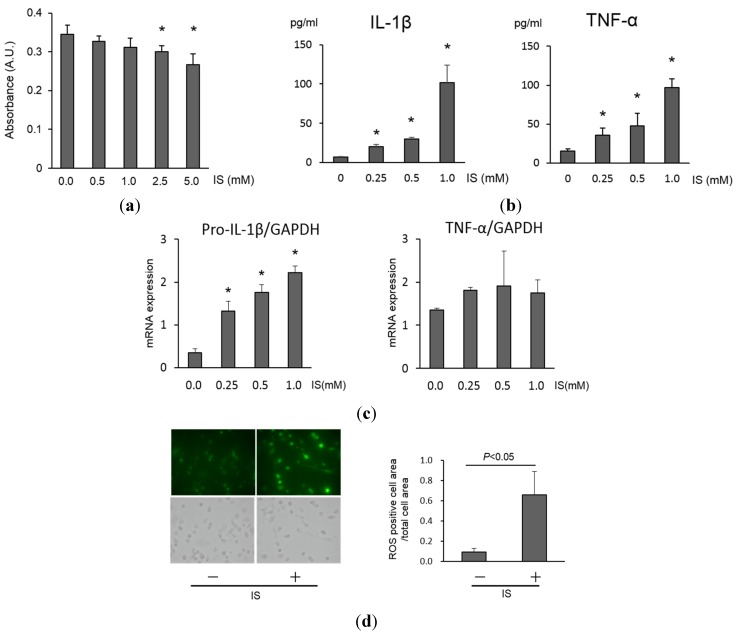
Cell viability, inflammation, and ROS production in macrophages exposed to IS. (**a**) Concentration-dependent effect of IS on THP-1 macrophage cell viability, examined using the MTS assay. Data represent the mean ± SD of three experiments. * *p* < 0.05 *vs.* 0 mM IS; (**b**) Concentration-dependent effect of IS on IL-1β and TNF-α production by THP-1 macrophages, as determined by ELISA. Data represent the mean ± SD of three experiments. * *p* < 0.05 *vs.* 0 mM IS; (**c**) Concentration-dependent effect of IS on pro-IL-1β and TNF-α mRNA expression in THP-1 macrophages. Data represent the mean ± SD of three experiments. * *p* < 0.05 *vs.* 0 mM IS; (**d**) The effect of IS on ROS production by THP-1 macrophages was examined by fluorescent microscopy. Data represent the mean ± SD of four experiments.

An MTS assay showed that exposure of THP-1 macrophages to IS at a concentration range of 0 to 1.0 mM did not affect their viability. However, IS at 2.5 and 5.0 mM significantly impaired the viability of macrophages (Figure 1a). IS increased the production of both interleukin-1β (IL-1β) and tumor necrosis factor-α (TNF-α) by THP-1 macrophages in a dose-dependent manner (Figure 1b). IS also significantly increased the mRNA expression of pro-IL-1β (Figure 1c) and its protein counterpart IL-1β (Figure S1a). Similarly, TNF-α expression also increased; however, the change was not statistically significant (Figure 1c). Comparable increases in the mRNA expression of IL-1β and TNF-α were also observed in human peripheral monocyte-derived macrophages (Figure S2a). When THP-1 macrophages were exposed to 1.0 mM of IS, reactive oxygen species (ROS) production increased significantly (0.66 ± 0.23 fluorescence/complete cell area *vs.* 0.09 ± 0.04 fluorescence/complete cell area without IS, *p* < 0.05, Figure 1d), compared to that in control cells. In addition, caspase-1 (50 kDa) expression as well as cleaved IL-1β decreased significantly in response to IS exposure, compared to that in the untreated control cells (Figure S3), which may show the consumption of caspase-1 after activation of inflammasome. These results indicate that IS at concentrations less than 1.0 mM promotes inflammatory reactions in macrophages, while higher concentrations directly impair macrophage viability.

### 2.2. Lipid Homeostasis

**Figure 2:**
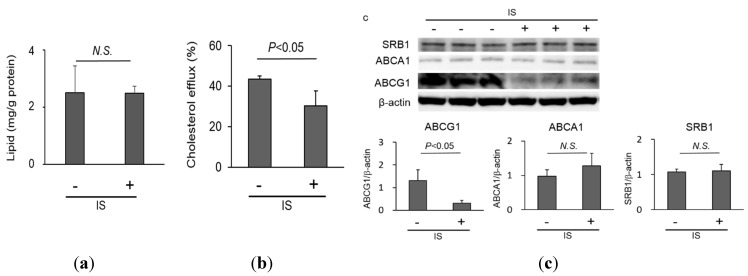
Lipid handling of macrophages exposed to indoxyl sulfate (IS). (**a**) Cholesterol uptake of macrophages. THP-1 macrophages were exposed to IS (1 mM) and acetylated low-density lipoprotein (50 µg/mL) for 8 h, and the cellular lipid contents, adjusted for protein concentration, were measured. Data represent the mean ± SD of four experiments; (**b**) Cholesterol efflux of macrophages. Lipid-enriched THP-1 macrophages were exposed to IS (1 mM) and high-density lipoprotein (50 µg/mL) from healthy subjects for 24 h, and the cellular lipid contents, adjusted for protein concentration, were measured. Data represent the mean ± SD of four experiments; (**c**) Western blot analysis of ATP-binding cassette transporters A1 (ABCA1), G1 (ABCG1), and scavenger receptor class B member1 (SRB1) protein expression. Data represent the mean ± SD of four experiments.

To determine the effect of IS on macrophage foam cell formation in atherosclerotic lesions, cellular lipid uptake and efflux reactions were examined. IS did not significantly influence the uptake of acetylated low-density lipoprotein (LDL) by THP-1 macrophages (2.49 ± 0.24 mg/g protein *vs.* 2.51 ± 0.93 mg/g protein without IS; Figure 2a). However, it significantly impaired macrophage cholesterol efflux induced by high-density lipoprotein (HDL) cholesterol in THP-1 macrophages (30.3% ± 7.3% *vs.* 43.5% ± 1.6% without IS, *p* < 0.05, Figure 2b) as well as human peripheral monocyte-derived macrophages (Figure S2b). We also examined the levels of ATP-binding cassette transporters A1 (ABCA1), G1 (ABCG1), and scavenger receptor class B member1 (SRB1) proteins, all of which are key lipid transporters for cholesterol efflux, in THP-1 macrophages exposed to IS. IS significantly decreased ABCG1 protein expression (0.31 ± 0.12 ABCG1/β-actin *vs.* 1.31 ± 0.49 ABCG1/β-actin without IS, *p* < 0.05, Figure 2c) but did not significantly affect ABCA1 (1.27 ± 0.37 ABCA1/β-actin *vs.* 0.98 ± 0.19 ABCA1/β-actin without IS, Figure 2c) and SRB1 (1.10 ± 0.18 SRB1/β-actin *vs.* 1.07 ± 0.08 SRB1/β-actin without IS, Figure 2c) protein expression. These results indicate that IS can impair macrophage cholesterol efflux and decrease ABCG1 protein expression.

### 2.3. Intervention with a Liver X Receptor Agonist for IS-Induced Macrophage Dysfunction

**Figure 3:**
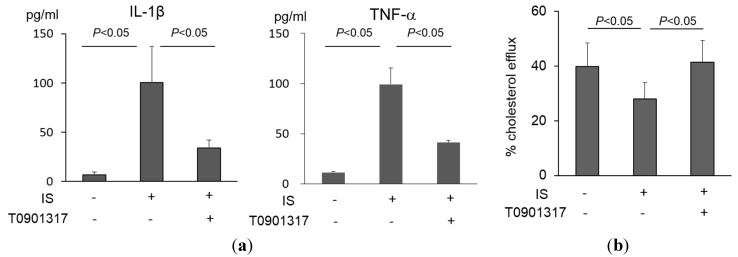
The effect of T0901317, a liver X receptor agonist, on macrophage function upon indoxyl sulfate (IS) exposure. (**a**) The effect of T0901317 on IL-1β and TNF-α production by THP-1 macrophages induced by IS. Inflammatory cytokine production was measured by ELISA. Data represent the mean ± SD of four experiments; (**b**) The effect of T0901317 on IS-induced impairment of macrophage cholesterol efflux. Data represent the mean ± SD of four experiments.

Liver X receptor (LXR), a member of the nuclear receptor family, regulates macrophage inflammation and cholesterol efflux [15]. The LXR agonist T0901317 ameliorated the macrophage inflammatory reaction (IL-1β; 34.0 ± 7.9 pg/mL *vs.* 100.3 ± 36.5 pg/mL without T0901317, *p* < 0.05, TNFα; 44.1 ± 2.2 pg/mL *vs.* 99.2 ± 16.4 pg/mL without T0901317, *p* < 0.05, Figure 3a) as well as cholesterol efflux (41.4 ± 7.8% *vs.* 27.9% ± 6.0% without T0901317, *p* < 0.05, Figure 3b). These results demonstrate that a LXR agonist can improve IS-induced macrophage inflammation as well as cholesterol efflux.

## 3. Discussion

The results of the present study demonstrate that IS, a protein-bound uremic toxin, can directly promote macrophage inflammation and impair lipid metabolism, and that these effects were ameliorated by treatment with an LXR agonist.

Uremic toxins such as IS and p-Cresyl sulfate (PCS) accumulate with increased production induced by CKD-related perturbation of the intestinal microbiome [16,17], as well as decreased excretion into urine. This suggests that the toxins directly affect cells in the atherosclerotic lesion and impair cellular function [12,18]. Macrophage foam cell formation within atherosclerotic lesions is a hallmark of CKD-induced acceleration of CVD, and it is important to understand the direct role of uremic toxins on macrophages.

In the present study, IS promoted IL-1β and TNF-α production by macrophages (Figure 1b,c) as well as ROS production (Figure 1d). In mouse models, subtotal nephrectomy induced acceleration of atherosclerosis both with deposition of IS and increased levels of inflammatory cytokines in the lesion [14]. Another study demonstrated that presence of pro-inflammatory macrophage phenotypes increased in the atherosclerosis lesion and were accelerated by renal dysfunction [19]. These results suggest that IS changes the macrophage phenotype to a pro-inflammatory state in atherosclerotic lesions. The dose of IS used to examine inflammation and lipid homeostasis in macrophages in this study was similar to the level in patients with CKD and those undergoing dialysis [13]. However, *in vivo*, IS has a high protein binding rate [20]. Higher doses of free IS compared with that in human serum is required for IS-induced THP-1 macrophage dysfunction, and it is possible that the amount of free IS in the atherosclerotic lesion is higher than that found in circulation. Previous studies have shown that the uremic milieu exerts toxicity on various cell types such as monocytes, leukocytes and tubular cells, which is related to vascular and renal disease progression [21,22,23,24,25,26,27,28]. For example, IS is known to induce cellular senescence and monocyte chemoattractant protein-1 expression mediated by the aryl hydrocarbon receptor (AhR) in human umbilical vein endothelial cells [26]. IS also downregulates Mas receptors in proximal tubular cells via the organic anion transporter 3, AhR, and the signal transducer and activator of transcription 3 [27]. Regarding macrophages, Adesso *et al.* reported that IS enhanced lipopolysaccharide-induced inflammatory reactions in J774A.1 murine monocyte macrophages [28].

The LXR agonist T0901317 modulated IS-induced macrophage inflammatory cytokine expression (Figure 3a). LXRs are cholesterol-sensing nuclear receptors that play a role in lipid metabolism but also have anti-inflammatory actions in macrophages [15]. LXR α and β knockout mice demonstrated increased atherosclerosis [29,30]. However, use of the LXR agonist inhibited acceleration of atherosclerosis [29]. An *in vitro* study showed that macrophages from LXR α and β knockout mice exhibited increased inducible nitric oxide synthase activity, whereas LXR agonists reduced this activity in wild type macrophages [31]. Taken together, IS may induce macrophage dysfunction through LXR-associated anti-inflammatory reactions, and modulation of the pathway may become a therapeutic strategy to inhibit CKD-induced acceleration of atherosclerosis.

Macrophage cholesterol efflux is one of the metrics of macrophage and HDL functionality *in vitro*. For example, HDL is less capable of promoting cholesterol efflux in patients who receive maintenance hemodialysis therapy [32,33]. Peritoneal macrophages from apolipoprotein E knockout mice with renal ablation showed less cholesterol efflux compared to those with intact kidneys [34]. The present data showed that macrophages reacted with IS and impaired cholesterol efflux induced by HDL in normal subjects (Figure 2b). The amount of cellular lipid uptake did not increase with exposure to IS (Figure 2a); thus, the mechanism of IS-induced macrophage foam cell formation can mainly be explained by reduced cholesterol efflux in the atherosclerotic lesion. Regarding the underlying mechanism of IS-induced impairment of macrophage lipid homeostasis, we found that expression of ABCG1 in macrophages, as opposed to ABCA1 and SRBI, decreased with exposure to IS (Figure 2c). Several interventions including protease inhibitors such as MG132, epoxomicin, and bortezomib can affect ABC transporters. These protease inhibitors enhanced both ABCA1 and ABCG1 expression, but not SRBI expression, in RAW264.7 macrophages, leading to an increase in cholesterol efflux [35]. Furthermore, sesamin also increased macrophage cholesterol efflux with increased ABCG1 expression. However, ABCA1 expression did not have the same effect [36]. Expression of ABC transporters is regulated by nuclear receptors such as LXR and peroxisome proliferator-activated receptor in macrophages. In this study, the results showed that activation of LXR by using T0901317 improved IS-induced impairment of cholesterol efflux (Figure 3b). A beneficial effect was observed even with exposure to uremic HDL [33]. This intervention can lead to inhibition of macrophage foam cell formation within atherosclerotic lesions in patients with CKD.

## 4. Experimental Section

### 4.1. Cell Culture

THP-1 human monocytic leukemia cells (American Type Culture Collection, Rockville, MD, USA) were cultured in RPMI 1640, supplemented with 10% FBS, 100 µg/mL penicillin, 100 µg/mL streptomycin, 10 mM HEPES, 1× MEM vitamin, and 0.5 µM 2-mercaptoethanol (Gibco-BRL, Gaithersburg, MD, USA). Cells were incubated at 37 °C in humidified air with 5% CO_2_. THP-1 cells at a density of 1 × 10^6^/mL were differentiated into macrophages by using 50 ng/mL phorbol 12-myristate 13-acetate (Sigma-Aldrich, St. Louis, MO, USA) for 72 h (THP-1 macrophages) [37]. Macrophage differentiation from monocytes was evidenced by their adherence to the culture plate [38]. Human monocyte-derived macrophages were also cultured according to previously published protocols [39].

### 4.2. Cell Viability *Assay*

The MTS assay was performed to assess the viability of cells reacted with IS (Sigma-Aldrich), using Cell Titer 96 Aqueous One Solution (Promega, Madison, WI, USA), according to the manufacturer’s protocol [40]. Briefly, 1 × 10^6^ THP-1 macrophages in 80 µL of medium on 96-well plates were exposed to IS (0–5.0 mM) for 24 h. MTS reagent (20 µL) was then added to cells, which were allowed to incubate at 37 °C for 1 h. Absorbance was measured at 492 nm using a Surise™ plate reader (TECAN Group Ltd, Männedorf, Switzerland).

### 4.3. Measurement of Inflammatory Cytokines in Medium

Macrophages were exposed to IS in FBS free medium at concentrations of 0–1.0 mM for 24 h. Cell supernatants were then collected, and the concentrations of IL-1β and TNF-α were measured using the human IL-1β (GEN-PROBE, San Diego, CA, USA) and TNF-α ELISA Kits (Life Technologies, Carlsbad, CA, USA), according to the manufacturer’s protocol.

### 4.4. Measurement of mRNA Expression

Macrophages were exposed to IS at a concentration of 0–1.0 mM for 24 h. Total RNA was extracted from cells by using the GenElute Mammalian Total RNA Miniprep Kit (Sigma-Aldrich) in accordance with the manufacturer’s instructions. Quantitative real-time PCR was performed using the One Step SYBR Plus RT PCR Kit on a Thermal Cycler Dice Real-time System (TP900, Takara, Otsu, Shiga, Japan). Primers for human pro-IL-1β (HA106116, Takara), TNF-α (HA072156 Takara), and GAPDH (HA067812, Takara) were used to quantify mRNA expression. GAPDH was used as an internal control.

### 4.5. Measurement of Reactive Oxygen Species Production

THP-1 macrophages were exposed to IS at concentrations of 1.0 mM for 24 h. ROS production was examined using the ROS/RNS Detection Kit (Enzo Life Sciences Farmingdale, NY, USA), according to the manufacturer’s instructions. Briefly, 2 × 10^5^ THP-1 macrophages on glass slides were reacted with the ROS/RNS 3-plex Detection Mix for 2 h. Cells were then exposed to IS (1.0 mM) for 30 min. Production of ROS was measured by calculating the fluorescent cell area divided by the complete cell area by fluorescence microscopy (BZ-8000 KEYENCE, Osaka, Japan).

### 4.6. Detection of Proteins in Cells

THP-1 macrophages were exposed to IS at concentrations of 1.0 mM for 24 h. Western blot analysis was performed to detect the expression of ABC transporters, IL-1β, and caspase-1 in cells. Whole-cell lysates were prepared in lysis buffer (20 mM Tris-HCl (pH 7.5), 150 mM NaCl, 500 mM EDTA, and 1% TritonX-100). The protein (18 µg) was separated on NuPAGE 4%–12% SDS-polyacrylamide gels (Life Technologies, Carlsbad, CA, USA), and transferred to a PVDF membrane. Membranes were blocked with 5% nonfat dry milk in TBST. To detect ABCA1, ABCG1, SRB1, IL-1β, and caspase-1, membranes were incubated overnight at 4 °C with monoclonal anti-ABCA1 antibodies (Abcam, Cambridge, MA, USA), polyclonal anti-ABCG1 antibodies (Novus Biologicals, Littleton, CO, USA), monoclonal anti-SRB1 antibodies (Abcam), polyclonal anti- IL-1β antibodies (Santa Cruz Biotechnology Inc., Dallas, TX, USA), and monoclonal anti-caspase-1 antibodies (Santa Cruz Biotechnology Inc.). To detect β-actin, membranes were incubated with anti β-actin antibodies (MBL, Nagoya, Aichi, Japan) for 30 min at room temperature. Horseradish peroxidase-linked secondary antibodies were used at a concentration of 1:5000 for ABCA1, 1:1000 for ABCG1, 1:1000 for SRB1, and signals were visualized as chemiluminescence (Lumi Vision PRO LPR-45/NP-1, TAITEC, Saitama, Japan). Chemiluminescence intensity was calculated using ImageJ.

### 4.7. Lipid Accumulation and Cholesterol Efflux Study

To study lipid uptake, THP-1 macrophages were loaded with acetylated LDL (50 µg/mL, Intracel, Frederick, MD, USA) for 8 h. Cells were washed with D-PBS 3 times and air-dried. Cellular lipid was extracted using 2-propanol overnight, and lipid content was quantified using the Cholesterol/Cholesteryl ester Quantitation Kit (Bio Vision, Milpitas, CA, USA) according to the manufacturer’s instructions. Cellular proteins were solubilized by addition of NaOH (0.1 N), and protein content was measured using the Bradford assay (Bio Rad, Hercules, CA, USA). Cholesterol uptake was determined as the increase in cellular cholesterol content at baseline *versus* the cholesterol content after reaction with acetylated LDL.

To study cholesterol efflux, macrophages were reacted with acetylated LDL (50 µg/mL) for 48 h. Lipid-rich macrophages were then reacted with HDL cholesterol (50 µg/mL) isolated from the plasma of healthy subjects using density-gradient ultracentrifugation for 24 h [33]. Cholesterol efflux was determined as the percentage decrease in cellular cholesterol content at baseline *versus* the cholesterol content after the reaction with HDL [33].

### 4.8. Reaction with a Liver X Receptor Agonist

THP-1 macrophages were exposed to the LXR agonist, T0901317 (Sigma-Aldrich) to intervene with IS induced disruption of macrophage function. THP-1 macrophages were exposed to IS (1 mM) and T0901317 (2 µM), and inflammatory cytokine production and cholesterol efflux were evaluated.

### 4.9. Statistics

Results are expressed as mean ± standard deviation. Statistical differences were assessed using the unpaired Student *t*-test or a single-factor analysis of variance followed by Bonferroni correction. *p <* 0.05 was considered significant.

## 5. Conclusions

IS directly promoted inflammatory reactions and impaired lipid homeostasis in macrophages, both of which were modulated by LXR agonists. These findings emphasize the importance of the interaction between uremic toxins and macrophages, which may be, at least in part, the underlying mechanism driving the acceleration of atherosclerosis in patients with CKD. In addition, this may also serve as a therapeutic target to ameliorate this condition.

## Supplementary Materials

Supplementary materials can be accessed at: http://www.mdpi.com/2072-6651/7/8/3155/s1.
