# Supplementary material for: Increased Proinflammatory Cytokine Production and Decreased Cholesterol Efflux Due to Downregulation of ABCG1 in Macrophages Exposed to Indoxyl Sulfate
Source: Toxins (Basel). 2015 Aug 14;7(8):3155–66. doi: 10.3390/toxins7083155 (PMC4549743; doi:10.3390/toxins7083155)
Supplement: Supplementary file 1 [file toxins-07-03155-s001.pdf]

Supplementary Materials

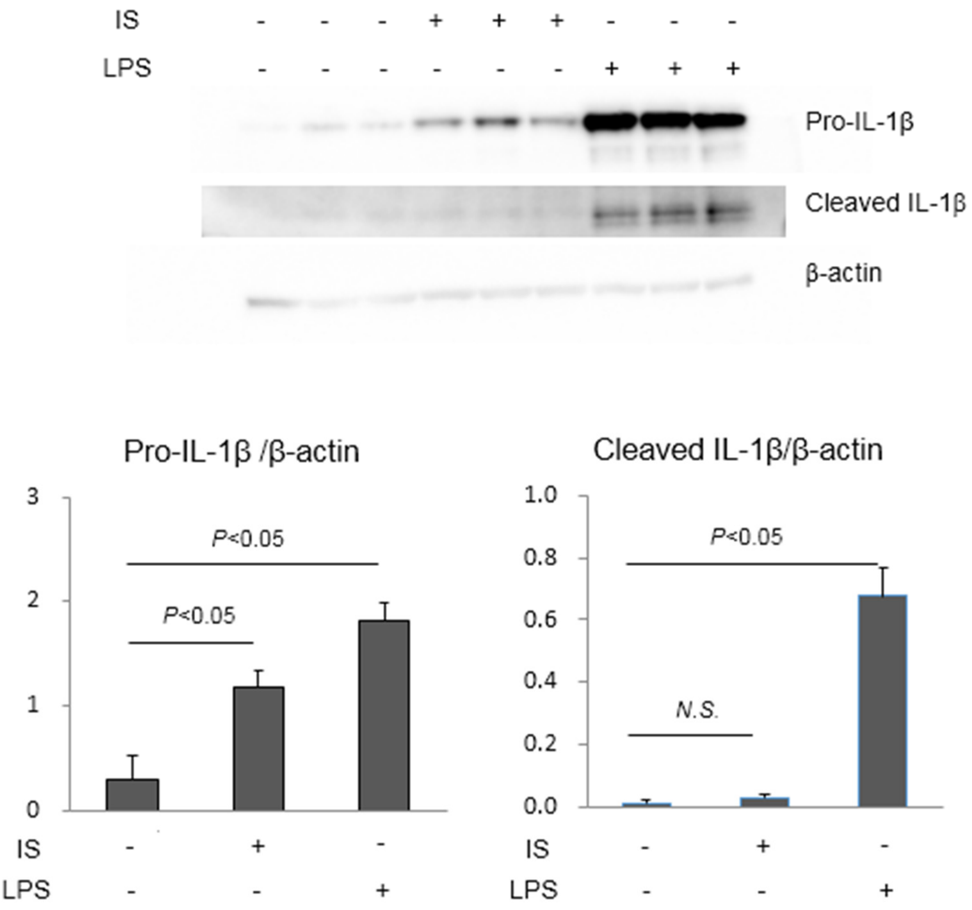

Figure S1. Western blot analysis evaluating IL-1b expression in THP-1 macrophages exposed to IS. Data represent the mean ± SD of three experiments.

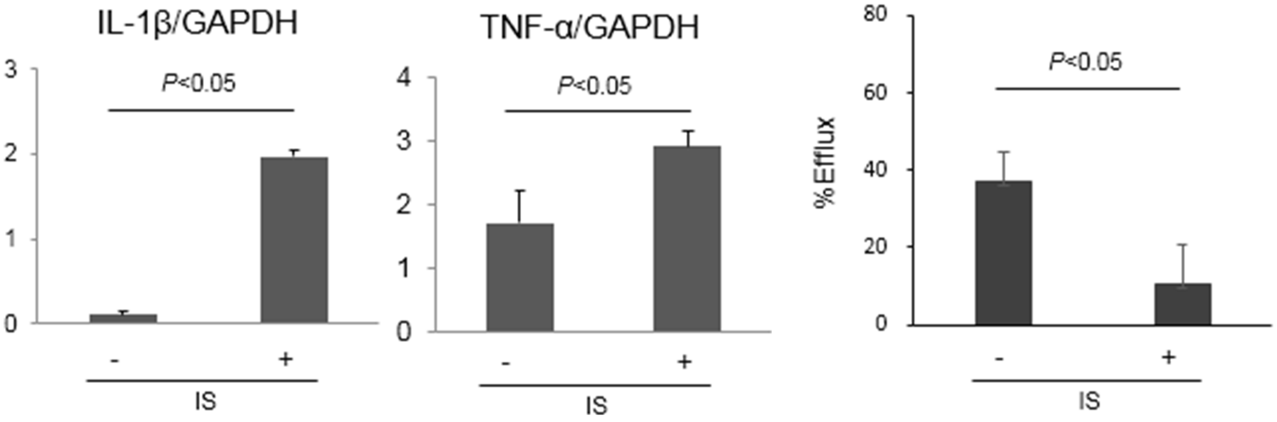

(**a**) (**b**)

Figure S2. (**a**) Effect of IS (1 mM) on pro-IL-1β and TNF-α mRNA expression in human peripheral monocyte-derived macrophages. Data represent the mean ± SD of three experiments; (**b**) Cholesterol efflux of macrophages. Lipid-enriched human peripheral monocyte-derived macrophages were exposed to IS (1 mM) and high-density lipoprotein (50 µg/mL) from healthy subjects for 24 h, and the cellular lipid contents adjusted for protein concentration were measured. Data represent the mean ± SD of three experiments.

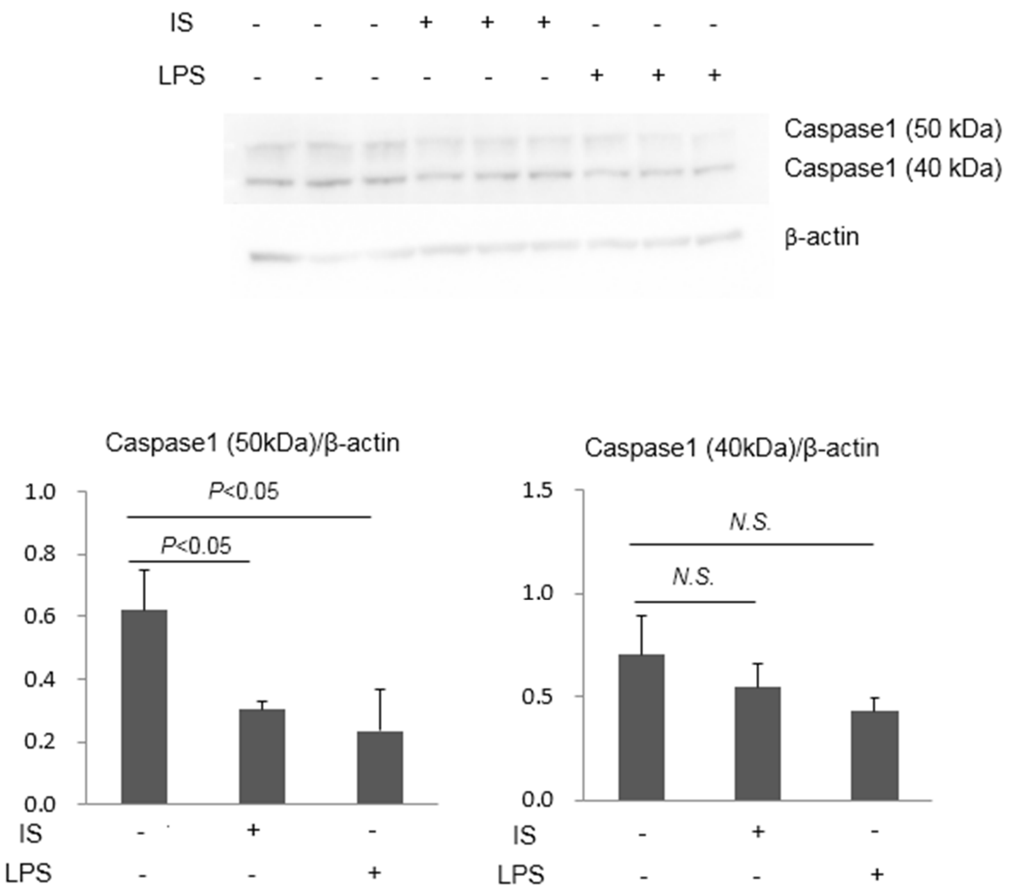

Figure S3. Western blot analysis of caspase-1 protein in THP-1 macrophages exposed to IS. Data represent the mean ± SD of three experiments.
